# Racial disparities in metastatic colorectal cancer outcomes revealed by tumor microbiome and transcriptome analysis with bevacizumab treatment

**DOI:** 10.3389/fphar.2023.1320028

**Published:** 2024-01-31

**Authors:** Lei Feng, Rui Wang, Qian Zhao, Jun Wang, Gang Luo, Chongwen Xu

**Affiliations:** ^1^ Department of Otorhinolaryngology-Head and Neck Surgery, The First Affiliated Hospital of Xi’an Jiaotong University, Xi’an, Shaanxi, China; ^2^ Department of Surgical Oncology, Hanzhong People’s Hospital, Hanzhong, Shaanxi, China; ^3^ Department of Thoracic Surgery, Cancer Centre, The First Affiliated Hospital of Xi’an Jiaotong University, Xi’an, Shaanxi, China; ^4^ Tongji Hospital Tongji Medical College of HUST, Wuhan, China

**Keywords:** mCRC, transcriptomics, intratumoral microbiome, multi-omics, racial variations, bevacizumab

## Abstract

**Background:** Metastatic colorectal cancer (mCRC) is a heterogeneous disease, often associated with poor outcomes and resistance to therapies. The racial variations in the molecular and microbiological profiles of mCRC patients, however, remain under-explored.

**Methods:** Using RNA-SEQ data, we extracted and analyzed actively transcribing microbiota within the tumor milieu, ensuring that the identified bacteria were not merely transient inhabitants but engaged in the tumor ecosystem. Also, we independently acquired samples from 12 mCRC patients, specifically, 6 White individuals and 6 of Black or African American descent. These samples underwent 16S rRNA sequencing.

**Results:** Our study revealed notable racial disparities in the molecular signatures and microbiota profiles of mCRC patients. The intersection of these data showcased the potential modulating effects of specific bacteria on gene expression. Particularly, the bacteria *Helicobacter* cinaedi and Sphingobium herbicidovorans emerged as significant influencers, with strong correlations to the genes SELENBP1 and SNORA38, respectively.

**Discussion:** These findings underscore the intricate interplay between host genomics and actively transcribing tumor microbiota in mCRC’s pathogenesis. The identified correlations between specific bacteria and genes highlight potential avenues for targeted therapies and a more personalized therapeutic approach.

## Introduction

Colorectal cancer, a global health challenge, ranks among the top causes of cancer-related deaths (Xi and Xu, 2021). Specifically, metastatic colorectal cancer (mCRC) represents a particularly aggressive form, often associated with poor prognosis (Mazzoli et al., 2022). Recent data indicate that while treatment strategies for mCRC have evolved, the median survival for advanced-stage patients remains suboptimal, hovering around 29 months (Lonardi et al., 2022). Amidst the therapeutic arsenal available for mCRC, Bevacizumab, an angiogenesis inhibitor targeting the vascular endothelial growth factor (VEGF), has emerged as a frontrunner (Chionh et al., 2022; de Rauglaudre et al., 2022). Clinical trials have demonstrated its efficacy in prolonging survival and improving response rates when combined with standard chemotherapy regimens (Fang et al., 2023; He et al., 2023).

As the era of personalized medicine takes center stage in oncology, it becomes increasingly clear that traditional, broad-spectrum approaches often fall short in addressing the unique genetic and molecular profiles of individual tumors (Pauli et al., 2017; LeSavage et al., 2022). The shift towards tailored therapies is rooted in the growing recognition of the complexity and diversity of the tumor microenvironment (Vitale et al., 2019). This intricate milieu, rich in cellular interactions and molecular crosstalk, is a testament to the dynamic nature of cancer (Lee and Schmitt, 2019). A conglomerate of stromal cells, immune cells, signaling molecules, and a diverse array of microorganisms, the tumor microenvironment stands at the crossroads of cancer progression, dictating not only the trajectory of tumor growth but also its susceptibility or resistance to treatments (Wu and Dai, 2017; Maacha et al., 2019). Pioneering investigations have cast a spotlight on the tumor microbiome, unraveling its deep-seated influence on cancer biology (Helmink et al., 2019; Wong-Rolle et al., 2021). These microbial communities, often specific to tumor types or even individual patients, have been linked to various aspects of cancer development, ranging from tumorigenesis to metastasis (Fu et al., 2022; Fu et al., 2023). More intriguingly, emerging evidence points to the microbiome’s role in modulating therapeutic responses, potentially by influencing drug metabolism, modulating the host immune response, or even directly interacting with therapeutic agents (Michaudel and Sokol, 2020; Nejman et al., 2020). Simultaneously, advances in genomics have ushered in the age of transcriptomics, providing unprecedented insights into the genetic orchestra that underpins tumor behavior (Tian et al., 2023). The tumor transcriptome, a real-time snapshot of gene expression, serves as a rich repository of information (Hunter et al., 2021). It not only charts the active genetic pathways within the tumor but also holds clues to potential points of vulnerabilities (Li et al., 2020). As such, transcriptomic analyses can unmask patterns of gene expressions that herald treatment resistances, or conversely, pinpoint genetic signatures predictive of therapeutic responsiveness (Casamassimi et al., 2017).

Nevertheless, while the intersections of the tumor microbiome and transcriptome with mCRC treatments have been explored, a significant blind spot remains: the influence of racial disparities (Carethers and Doubeni, 2020). It’s well-documented that racial and ethnic differences can drastically affect disease outcomes (de Klerk et al., 2018). For instance, African Americans with colorectal cancer have a 20% higher incidence rate and a 40% higher mortality rate compared to their Caucasian counterparts (Cobb et al., 2022). Such disparities could arise from a confluence of genetic variations, environmental exposures, and even socio-economic factors (Zaki et al., 2023). However, the specific molecular and microbial underpinnings, especially in the context of mCRC and Bevacizumab treatment, remain largely uncharted.

In this comprehensive study, we endeavor to bridge this knowledge gap. We aim to dissect the interplay of racial disparities with the tumor microbiome and transcriptome in mCRC patients undergoing Bevacizumab treatment. By unraveling the microbial and genetic nuances specific to different racial groups, we aspire to pave the way for more tailored therapeutic strategies, ensuring that the promise of personalized medicine is extended across all racial and ethnic divides.

## Materials and methods

### Tumor microbiome profiling through RNA sequencing

To delve deeper into the tumor microbiome, we embarked on an analytical journey using high-throughput RNA sequencing (RNA-seq) data, which was obtained from the renowned public repository, GEO, under the accession number GSE196576 (Innocenti et al., 2022). The initial step in our analytical pipeline emphasized the significance of data quality. Thus, we applied FastQC, a widely recognized quality control tool for high throughput sequence data, ensuring that our RNA-seq reads were of optimal quality for subsequent analyses. Recognizing the potential interference of host-associated reads in our microbial profiling, it was imperative to segregate them. This was adeptly accomplished using Samtools, a suite that’s pivotal for intricate interactions with high-throughput sequencing data. With the host reads meticulously filtered out, our focus transitioned to the crux of our analysis—taxonomic classification. For this intricate task, we employed Kraken2. Renowned for its unparalleled precision and efficiency in metagenomic classification, Kraken2 provided the granularity we sought in our taxonomic assignments.

### Transcriptome data processing using the nf-core/rnaseq pipeline

In our quest to unravel the intricacies of the tumor transcriptome, we leveraged the prowess of the “nf-core/rnaseq” pipeline, a cutting-edge framework meticulously curated by the nextflow community. This pipeline is not just a mere tool but an embodiment of state-of-the-art practices in RNA-seq data processing, harmonizing multiple essential processes into a cohesive workflow. The “nf-core/rnaseq” pipeline commences with quality control checks on raw sequencing data using FastQC, ensuring the data’s integrity. It then proceeds to read trimming, leveraging the capabilities of Trimmomatic to remove any adapters or low-quality sequences, ensuring only high-fidelity reads are retained for downstream processes. The trimmed reads are subsequently aligned to the reference genome using the STAR aligner, a tool celebrated for its speed and accuracy in RNA-seq read mapping. Post alignment, featureCounts tallies the number of reads associated with each gene, enabling a quantitative overview of gene expression. Moreover, the pipeline integrates various quality control metrics post-alignment using tools like RSeQC, ensuring that the resultant data remains of the highest caliber for downstream analyses.

### Clinical sample collection

Clinical tissue samples were meticulously collected from patients diagnosed with metastatic colorectal cancer (mCRC). A total of 18 mCRC patients were enrolled in this study, with six individuals identifying as White and six as Black or African American and six Asian. Informed consent was obtained from all participants prior to sample collection, following the ethical guidelines set by the Institutional Review Board (IRB). Tissue samples were procured during surgical resection of primary tumors. The collected tissues were immediately snap-frozen in liquid nitrogen and stored at −80°C until further analysis to preserve the RNA and microbial integrity. Patient data, including age, sex, race, and clinical outcomes, were anonymized and recorded. Through this standardized and ethically compliant process, we ensured the reliability and validity of the clinical samples used in our study.

### 16S rRNA sequencing

To meticulously decipher the microbiota landscape within mCRC tissues, we employed 16S rRNA gene sequencing, a gold-standard technique for studying microbial communities present within biological samples. Tissue samples were diligently collected from 12 patients diagnosed with mCRC, with six identifying as White and the remaining six as Black or African American. Each sample was immediately snap-frozen to preserve the integrity of the microbial DNA. Subsequently, microbial DNA was extracted using a PureLink™ Microbiome DNA Purification Kit (Thermo Fisher, United States), adhering strictly to the manufacturer’s protocol to ensure consistency and reliability in the extracted genetic material. The V3-V4 hypervariable regions of the 16S rRNA gene were amplified using universal primers; V3-V4 PRIMER-F 5′-CCTACGGRRBGCASCAGKVRVGAAT-3′and PRIMER -R 5′-GGACTACNVGGGTWTCTAATCC-3′. The PCR reaction was meticulously optimized to produce reliable and reproducible results. Upon completion of the PCR process, the amplified products were verified through agarose gel electrophoresis. Thereafter, the PCR products were purified, quantified, and pooled equimolarly for sequencing. The pooled samples were then sequenced on an Illumina MiSeq platform, utilizing a 2 × 300 bp paired-end configuration, thereby generating comprehensive and high-resolution data of the microbial communities present within each sample.

### RT-PCR

Total RNA was extracted from the CRC tissue samples using the RNeasy Mini Kit (Qiagen, Hilden, Germany), following the manufacturer’s protocol. The quality and concentration of the isolated RNA were meticulously assessed using the NanoDrop ND-1000 spectrophotometer (NanoDrop Technologies, Wilmington, DE, United States). Quantitative PCR was performed using the Power SYBR Green PCR Master Mix (Applied Biosystems, Foster City, CA, United States) on a 7,500 Fast Real-Time PCR System (Applied Biosystems). Primers for the target genes, SELENBP1 and SNORA38, and the housekeeping gene GAPDH were designed using the Primer Express Software (Applied Biosystems). The specific primer sequences of SELENBP1 forward: 5′-ACC​CAG​GGA​AGA​GAT​CGT​CTA-3′, reverse: 5′-ACT​TGG​GGT​CAA​CAT​CCA​CAG-3′; SNORA38 forward: 5′-CGT​GTC​TGT​GGT​TCC​CTG​TC-3′, reverse: 5′- AGC​AAG​CTG​GCC​TCA​AAG​TT-3′; GAPDH forward: 5′-CAT​GTA​CGT​TGC​TAT​CCA​GGC-3′, reverse: 5′-CTC​CTT​AAT​GTC​ACG​CAC​GAT-3′. Each reaction was conducted in triplicate, with the mean value used for further analysis. The thermal cycling conditions were as follows: initial denaturation at 95°C for 10 min, followed by 40 cycles of denaturation at 95°C for 15 s, and annealing and extension at 60°C for 1 min.

### Revisiting the clinical trial

In the vast tapestry of the CALGB/SWOG 80405 trial, our present endeavor narrows its gaze on two specific regimens: bevacizumab and the synergistic cetuximab/bevacizumab combination. This refined focus stems from the quest to unravel the intricacies of these treatments in a more granular context. To discern the interplay of race and its potential influence on clinical outcomes, bridging the gap between genetics and therapeutic efficacy. Our investigation seeks to disentangle the nuanced relationship between diverse racial backgrounds and their respective clinical trajectories. By delving deep into the databank of the trial, we meticulously sift through patient demographics, juxtaposing them against an array of clinical parameters (Table 1). This rigorous exploration is not just an academic exercise but an attempt to unmask the subtle, often overlooked racial disparities that might modulate treatment responses. While the original CALGB/SWOG 80405 trial offered a broad panorama, our analysis is akin to a magnifying glass, emphasizing the details, drawing correlations, and aiming to enhance the personalized medicine paradigm. With the guiding light of trial identifier NCT00265850, we embark on this journey to understand better the racial tapestry in the context of bevacizumab and cetuximab/bevacizumab treatments.

**TABLE 1:** Comprehensive clinical demographics and characteristics by ethnicity.

Characteristic/Attribute	Black or African American	White	Asian	Not reported
Total patients	33	314	7	5
Age (years)				
Median	54	61.5	57	57
Range	(32–79)	(24–82)	(25–74)	(50–70)
Gender				
Female	11	125	3	1
Male	22	189	4	4
Progression free survival time (months)				
Median	8.9	8.7	11.1	11.9
Follow up time (months)				
Median	22.7	22.2	16.9	26.3
ECOG performance status				
Median	1	1	1	0
Number of metastatic sites				
Liver (Median)	1	1	1	1
Adjuvant. Chemotherapy				
YES	10	121	3	2
NO	23	193	4	3
Pelvic. Radiation				
YES	2	32	0	0
NO	31	282	7	5
KRAS				
wt	10	116	—	1
mut	7	33	—	
Unknown	16	165	7	4
NRAS				
wt	17	144	—	1
mut	—	5	—	—
Unknown	16	165	7	4
MSI status				
MSS	13	128	—	1
MSI-H	2	12	—	—
MSI-L	2	6	—	—
Unknown	16	168	7	4
Side				
Right	8	80	—	1
Left	8	55	—	—
Transverse	—	7	—	—
Unknown	17	172	7	4

### Statistical analysis and data interpretation

Navigating the complex interplay between transcriptomics, the tumor microbiome, and racial disparities in metastatic colorectal cancer necessitated a robust and comprehensive statistical framework. Our analytical journey commenced with data acquisition from the public repository GSE196576, part of the GEO database. For differential expression analysis, we leveraged Python’s “scipy.stats” library to perform the Student’s *t*-test, ensuring a rigorous identification of genes with notable expression differences. The threshold of significance was set based on an adjusted *p*-value, incorporating the Benjamini–Hochberg correction, and was set at less than 0.05. All analyses were conducted using Python version 3.10. The microbial dimension of our study called for both alpha and beta diversity analyses. While alpha diversity provided a lens into the richness and evenness of microbial entities within individual samples, beta diversity was instrumental in highlighting the compositional variations between samples, thus elucidating intergroup differences. For correlation assessments, the Pearson correlation coefficients, derived using Python’s “scipy” and “pandas” libraries, served as a beacon, revealing linear associations between specific microbiota and gene expression levels. Notably, only correlations with an absolute value of (|r| > 0.5) and a *p*-value below 0.05 were deemed significant. Survival patterns, central to understanding treatment efficacy, were dissected using Kaplan-Meier survival plots generated via the “lifelines” Python package. Distinctions between survival curves underwent rigorous statistical scrutiny via the log-rank test, while multivariate Cox regression analyses fine-tuned our understanding, bringing potential confounders into the analytical fold and pinpointing independent predictors of outcomes. The art of data visualization was paramount. With Python’s “matplotlib” and “seaborn” libraries at our disposal, we crafted insightful visual representations, spanning from heatmaps to boxplots. Given the dimensionality of our transcriptomic data and the plethora of tests, the Benjamini–Hochberg procedure was indispensable in controlling the false discovery rate, cementing the statistical reliability of our findings.

## Results

### Ethnicity-centric clinical and genetic analysis of patients

**FIGURE 1:**
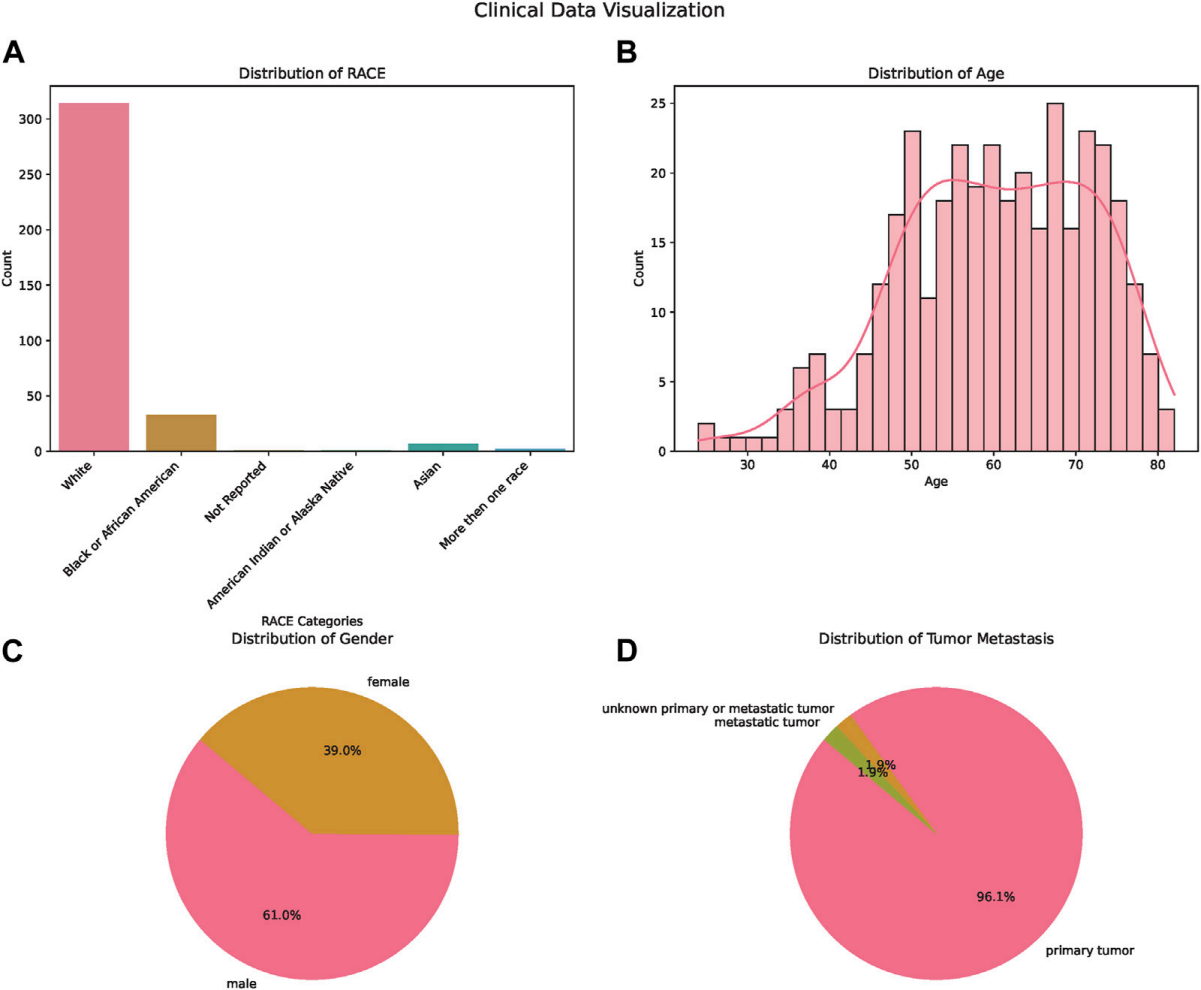
Demographic and Clinical Characteristics of the Study Population. **(A)** Bar chart illustrating the distribution of patients across different ethnicities: White, Asian, Black or African American, and American Indian or Alaska Native. **(B)** Bar chart displaying age distribution of the patients, providing insights into the age range and median age of the participants. **(C)** Pie chart showcasing the gender distribution of the study participants, highlighting the proportions of male and female patients. **(D)** Pie chart depicting the tumor metastasis status among the patients, signifying the proportion of patients with and without tumor metastasis.

In our comprehensive analysis of patient demographics and clinical characteristics stratified by ethnicity, we observed distinct patterns (Table 1). Most of the participants were of White descent (314), followed by Black or African American (33), Asian (7), with 5 individuals not reporting their ethnicity (Figure 1A). For age, White participants presented the highest median age at 61.5 years, while Black or African American and Not Reported groups both shared a median age of 57 years. The age ranges across the ethnicities varied, with the White group demonstrating the broadest span from 24 to 82 years (Figure 1B). Regarding gender distribution, males predominated in all ethnic groups except for the Asian cohort, where the ratio was almost equal. Specifically, the White group consisted of 189 males and 125 females (Figure 1C). When analyzing the Progression-Free Survival Time, Asian participants exhibited the longest median duration of 11.1 months. In contrast, the White group had a median of 8.7 months, slightly less than the Black or African American group’s 8.9 months. Follow-up durations were relatively consistent across groups, with the Not Reported group having the longest median follow-up time at 26.3 months. The ECOG Performance Status was generally consistent across the groups, with a median score of 1, except for the Not Reported group, which had a median score of 0. Genetic analyses revealed that a notable proportion of the White group exhibited wild-type KRAS (116) and NRAS (144). MSI status predominantly showed MSS phenotype in most groups where data was available. Tumor location displayed varied distributions across ethnicities, with the right side being the most common location in the Black or African American and White cohorts (Figure 1D).

### Diversity of tumor microbiome across ethnic groups

**FIGURE 2:**
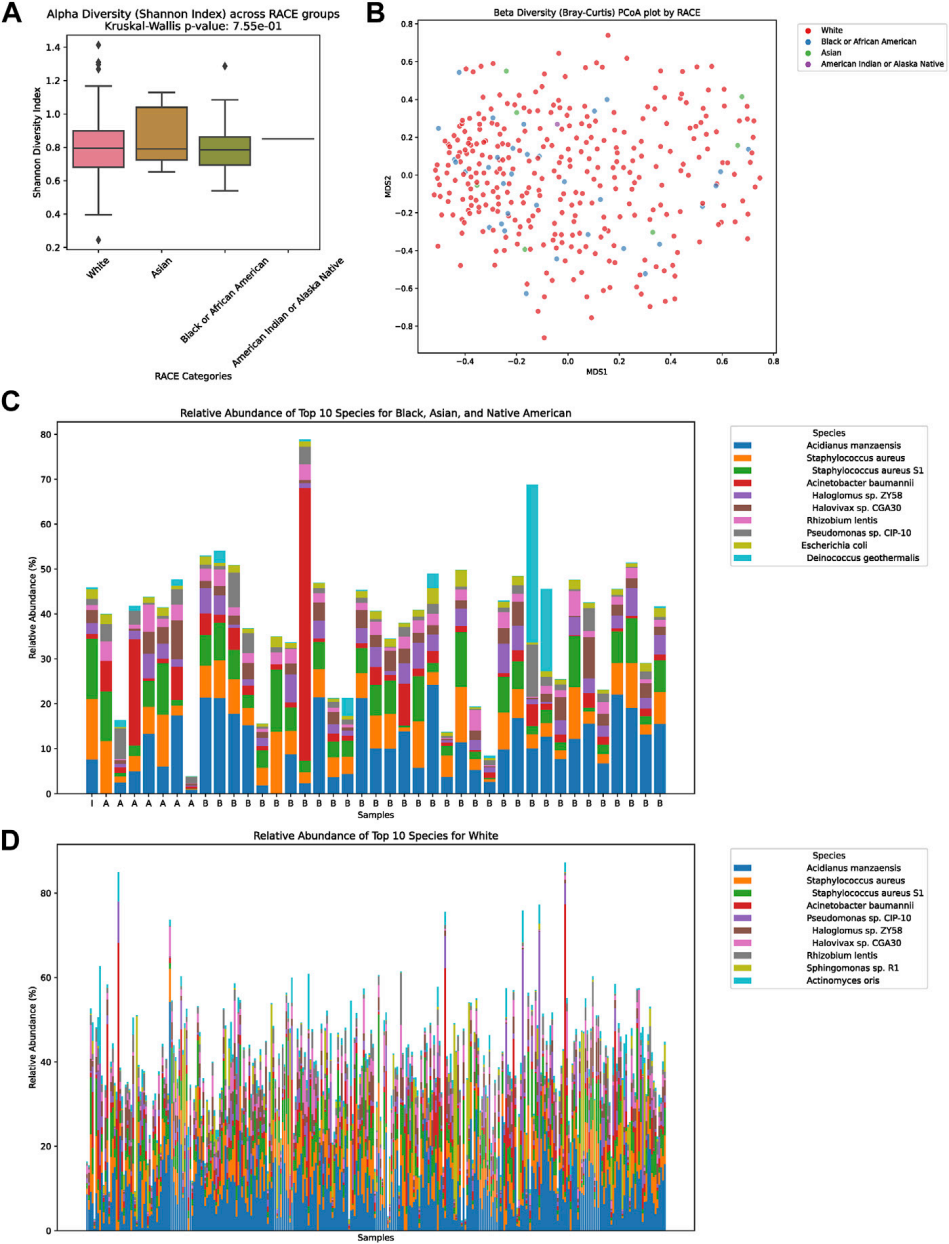
Diversity and Composition of the Tumor Microbiome Across Ethnic Groups. **(A)** Alpha diversity representation across the four ethnic groups: White, Asian, Black or African American, and American Indian or Alaska Native. No significant differences were observed among the groups (*p* = 0.755). **(B)** Beta diversity illustrated via a PCoA plot, showing microbial community differences between samples among the ethnicities. **(C)** Relative abundance of the top ten microbial species for the combined group of Asian, Black or African American, and American Indian or Alaska Native. The dominant species in this group were Acidianus manzaensis, *Staphylococcus aureus*, *Staphylococcus aureus* S1, *Acinetobacter* baumannii, Haloglomus sp. ZY58, Halovivax sp. CGA30, Rhizobium lentis, *Pseudomonas* sp. CIP-10, *Escherichia coli*, and Deinococcus geothermalis. **(D)** Relative abundance of the top ten microbial species for the White ethnic group. The predominant species for this group were Acidianus manzaensis, *Staphylococcus aureus*, *Staphylococcus aureus* S1, *Acinetobacter* baumannii, *Pseudomonas* sp. CIP-10, Haloglomus sp. ZY58, Halovivax sp. CGA30, Rhizobium lentis, Sphingomonas sp. R1, and *Actinomyces* oris.

In our detailed investigation into the tumor microbiome diversity across various ethnicities, we uncovered consistent patterns. The alpha diversity, which represents the variety of species in individual samples, displayed no significant variations among the White, Asian, Black or African American, and American Indian or Alaska Native groups (*p* = 0.755, Figure 2A). Similarly, the beta diversity, which underscores the microbial community differences between samples, also showed no pronounced distinction among the ethnicities, as illustrated in the PCoA plot (Figure 2B). Taking a closer look at the relative abundances of microbial species, for the combined group of Asian, Black or African American, and American Indian or Alaska Native, the top ten species were Acidianus manzaensis, *Staphylococcus aureus*, *S. aureus* S1, *Acinetobacter* baumannii, Haloglomus sp. ZY58, Halovivax sp. CGA30, Rhizobium lentis, *Pseudomonas* sp. CIP-10, *Escherichia coli*, and Deinococcus geothermalis (Figure 2C). On the other hand, for White individuals, the ten predominant species were Acidianus manzaensis, *S. aureus*, *S. aureus* S1, *Acinetobacter* baumannii, *Pseudomonas* sp. CIP-10, Haloglomus sp. ZY58, Halovivax sp. CGA30, Rhizobium lentis, Sphingomonas sp. R1, and *Actinomyces* oris (Figure 2D).

### Differential microbial abundance across ethnicities

**FIGURE 3:**
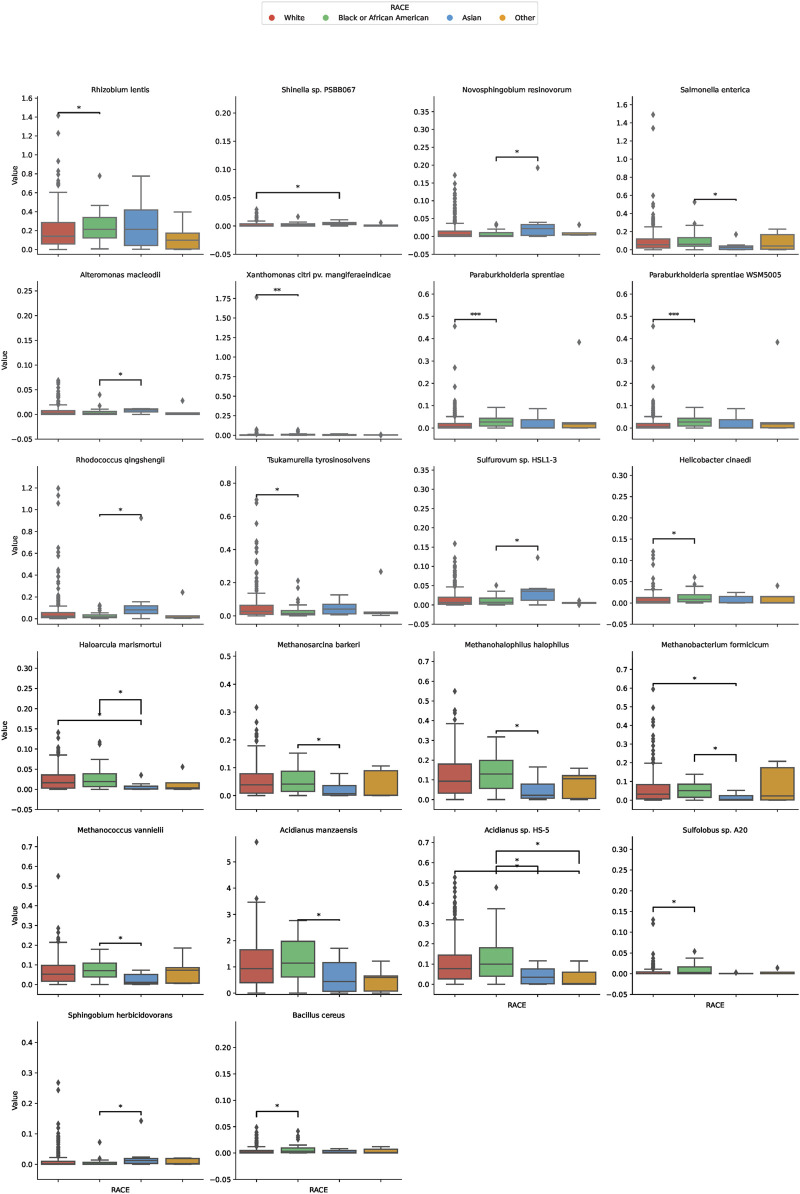
Differential Microbial Abundance Across Ethnicities. The boxplots in this figure depict the variation in microbial abundance for specific species across diverse ethnic groups. Each color represents a distinct ethnicity: red for White, green for Black or African American, blue for Asian, and yellow for Others. *, *p* < 0.05; **, *p* < 0.01.

In our comprehensive assessment of microbial diversity across different ethnic groups, significant variations were identified in the abundance of specific species (Figure 3). These disparities were especially pronounced when comparing the White and Black groups, as well as between the White and Asian cohorts, and the Black and Asian cohorts. Among the White and Black cohorts, species such as *Tsukamurella* tyrosinosolvens and *Helicobacter* cinaediwere found to be more abundant in the White group, with fold changes of 1.79 and 0.40, respectively. On the contrary, Sulfolobus sp. A20 showed a substantial decrease in abundance in the White group with a fold change of 0.081. For the White and Asian cohorts, Haloarcula marismortui and Methanobacterium formicicum were notably more abundant in the White population, with fold changes of 23.16 and 8.98, respectively. However, Shinella sp. PSBB067 demonstrated a decreased presence in the White group, registering a fold change of 0.166. Comparing the Black and Asian groups, there was an overwhelming abundance of Haloarcula marismortui and Methanobacterium formicicum in the Black cohort, with fold changes of 26.88 and 14.34, respectively. In contrast, Sphingobium herbicidovorans recorded a diminished presence in the Black group with a fold change of 0.074. The comprehensive list of microbial species and their fold changes across ethnic groups can be found in Supplementary Table S1.

### Prognostic implications of differential microbial abundance

**FIGURE 4:**
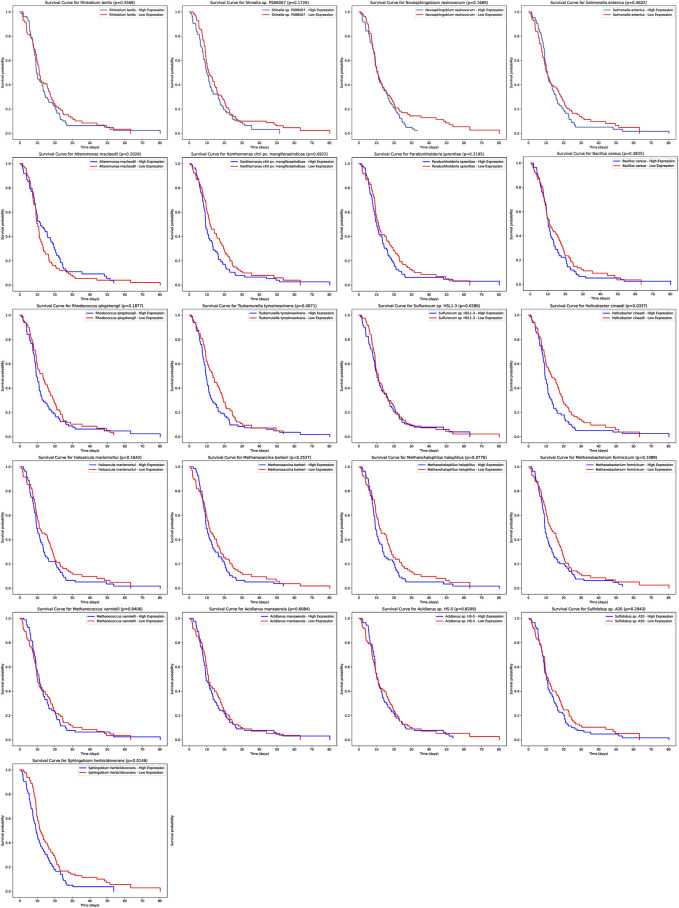
Prognostic Significance of Microbial Abundance in Tumor Samples. The survival curves depict the progression-free survival (PFS) based on the expression of two microbial species. Elevated levels of *Helicobacter* cinaedi and Sphingobium herbicidovorans were associated with poorer survival outcomes, with *p*-values of 0.0337 and 0.0146 respectively.

Our analysis extended to understanding the potential prognostic implications of the microbial abundance in tumor samples (Figure 4). Of particular interest, two microbial species demonstrated a significant association with progression-free survival (PFS). Elevated expression of *Helicobacter* cinaedi was associated with a poorer survival outcome, as evidenced by a *p*-value of 0.0337. Similarly, higher levels of Sphingobium herbicidovorans also indicated a worse prognosis with a *p*-value of 0.0146. These findings underscore the potential prognostic value of specific microbial species within tumor samples and warrant further exploration into their role in patient outcomes.

### Dissecting racial disparities: differentially expressed genes and their interplay with tumoral microbiota

**FIGURE 5:**
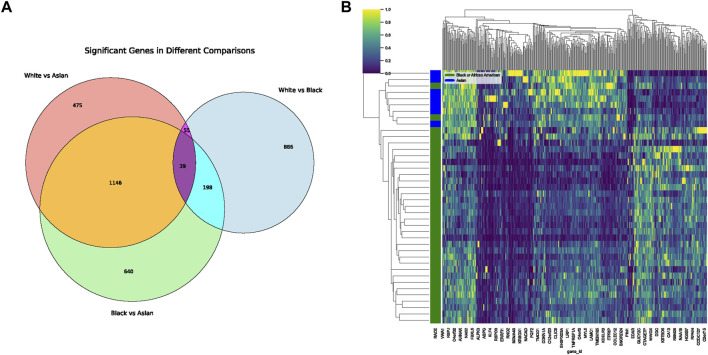
Differential Gene Expression Across Racial Groups. **(A)** Venn diagram displaying the overlap of differentially expressed genes among White, Black, and Asian populations. The shared and unique gene expressions are represented in their respective intersections. **(B)** Heatmap representing differentially expressed genes unique to the Black vs. Asian comparison. Gene details can be found in Supplementary Table S2.

**FIGURE 6:**
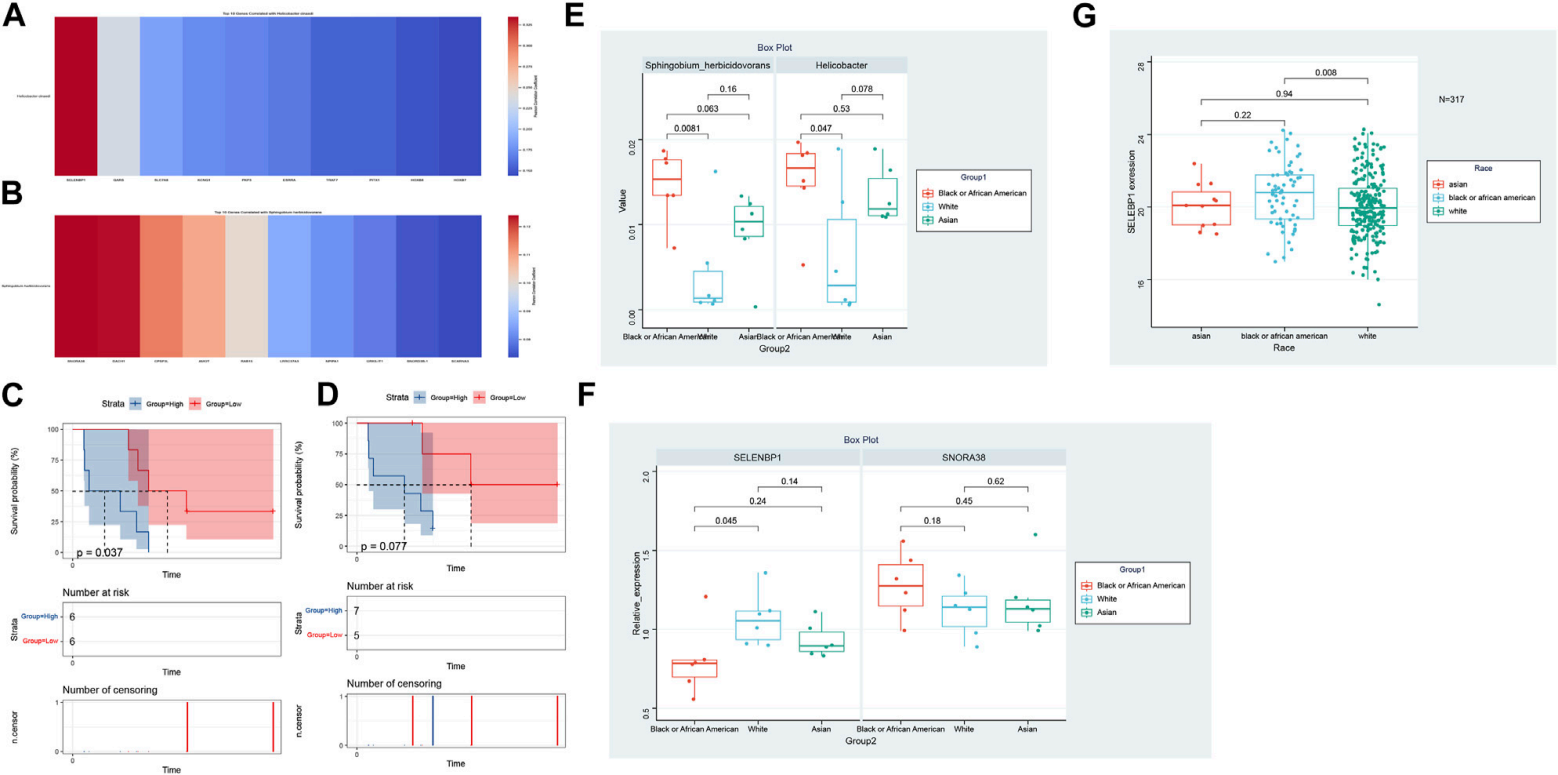
Genomic-Microbiota Associations in Tumors. **(A)** Top 10 genes exhibiting significant correlation with *Helicobacter* cinaedi. The most notably associated gene in this set is SELENBP1. **(B)** Top 10 genes displaying pronounced correlation with Sphingobium herbicidovorans Among these, SNORA38 emerges as the gene with the highest significance. **(C)** Kaplan-Meier Survival Analysis of Patients with Varied Sphingobium herbicidovorans Expression Levels. The figure illustrates the survival curves of mCRC patients with low (red line) versus high (blue line) expression levels of Sphingobium herbicidovorans. A statistically significant improvement in patient survival is observed with lower expression levels of this bacterial strain, as evidenced by a *p*-value of <0.05. **(D)** Survival Analysis of Patients Based on *Helicobacter* cinaedi Expression Levels. The Kaplan-Meier survival curves for patients with low (red line) and high (blue line) expression levels of *Helicobacter* cinaedi are depicted. While there isn't a statistically significant difference in survival between the two groups (*p* = 0.07), a noticeable trend of separation between the curves suggests potential implications of *Helicobacter* cinaedi expression levels on patient survival. **(E)** Differential Expression of Sphingobium herbicidovorans and *Helicobacter* cinaedi in Racial Groups. This figure demonstrates the upregulation of Sphingobium herbicidovorans and *Helicobacter* cinaedi in Black or African American mCRC patients compared to their Caucasian counterparts. The Asian group shows no significant difference in the expression levels of these bacterial strains compared to both Black or African American and Caucasian groups. Each bar represents the average expression level of the respective bacterial strain in each racial group, with error bars indicating the standard deviation. **(F)** Expression Analysis of SELENBP1 and SNORA38 in Different Racial Groups. The bar graph illustrates the expression levels of SELENBP1 and SNORA38 in Caucasian and Black or African American mCRC patients, with significant differences noted in SELENBP1 expression between these two groups. The addition of the Asian group shows that there are no significant differences in the expression of SELENBP1 and SNORA38 when compared to both the Caucasian and Black or African American groups. Error bars represent standard deviation. **(G)** Expression Analysis of SELENBP1 in Different Racial Groups of TCGA (COAD).

In our comprehensive exploration of the racial differences in gene expression and their potential interaction with the tumor microbiome, we observed striking contrasts. Figure 5A presents a Venn diagram detailing the overlap of differentially expressed genes between the three racial groups. Remarkably, 39 genes were commonly differentially expressed across all pairwise comparisons. However, exclusive gene expression patterns also emerged: 886 genes were uniquely altered between White and Black populations, 640 genes between Black and Asian, and 475 genes between White and Asian. The heatmaps in Figure 5B, delve deeper, visualizing these differentially expressed genes for the Black vs. Asian, White vs. Asian, and White vs. Black comparisons, respectively. For in-depth gene details and annotations, we refer readers to Supplementary Tables S2–S4. Transitioning from the genomic landscape to its interplay with the microbiome, we analyzed the association between these racially differentiated genes and the two microbial species previously identified to be prognostically significant. Figure 6A showcases the top 10 genes correlated with *Helicobacter* cinaedi. Among these, SELENBP1 emerged as the most significantly associated gene. Similarly, Figure 6B highlights the top 10 genes correlated with Sphingobium herbicidovorans, with SNORA38 standing out as the most notable. Upon meticulous examination of the independently collected PFS survival data from 12 patients, it is evident that lower expression levels of Sphingobium herbicidovorans correlate with improved survival rates, as substantiated by a *p*-value less than 0.05 (Figure 6C). Conversely, while no significant disparity in survival rates is observed between low and high expression levels of *Helicobacter* cinaedi (*p* = 0.07), a conspicuous divergence trend between the two expression levels is noticeable (Figure 6D; Supplementary Figure S1D). Additionally, an in-depth analysis revealed a significant upregulation of both Sphingobium herbicidovorans and *Helicobacter* cinaedi in the Black or African American patient group (Figure 6E; Supplementary Figure S1E). In synchrony with these findings, the expressions of SELENBP1 and SNORA38—which are correlated with the respective bacterial strains—were also validated. Remarkably, the expression of SELENBP1 is significantly reduced in the Black or African American group, as depicted in Figure 6F. On the other hand, no significant difference was observed in the expression levels of SNORA38 between the groups. According to the comprehensive TCGA (COAD) dataset analysis, it was observed that SELEBP1 expression levels were the lowest in White individuals and highest in Black or African American individuals, denoting a significant disparity (Figure 6G). Intriguingly, the expression levels in Asian individuals were intermediate, showing no significant differences when compared to either group, in a study encompassing 317 subjects (Asians = 11, Black or African American = 65, and White = 241).

## Discussion

Metastatic colorectal cancer (mCRC) remains a significant clinical challenge, with its heterogeneity and adaptability often leading to therapy resistance and dismal outcomes (Xi and Xu, 2021; Mazzoli et al., 2022). Bevacizumab, an angiogenesis inhibitor, has emerged as a promising therapeutic agent in the treatment of mCRC (Garcia et al., 2020). By targeting vascular endothelial growth factor (VEGF), Bevacizumab reduces tumor blood supply, making it a cornerstone in the current mCRC treatment paradigm (Chionh et al., 2022; de Rauglaudre et al., 2022). A distinguishing feature of our study lies in the methodological approach of extracting microbiota data from RNA-SEQ. Unlike previous endeavors that sourced microbial abundance data from genomic sequences, our approach ensured that the microbial data we analyzed represented bacteria actively transcribing within the tumor milieu. The utilization of RNA-seq derived data for the training set and DNA-based 16S rRNA sequencing for validation indeed introduces methodological nuances. While RNA-seq offers insights into the active microbial community by capturing expressed genes, 16S rRNA sequencing identifies the broader microbial composition. The potential discrepancy between these methodologies underscores the importance of interpreting results within the context of the chosen method. Though each approach has its strengths, their combined use in our study seeks to provide a comprehensive view of the microbial landscape, with RNA-seq highlighting functional dynamics and 16S rRNA offering a snapshot of overall microbial diversity. Essentially, this means that the bacteria identified are not mere transient inhabitants but are actively participating in the tumor ecosystem, potentially influencing tumor behavior and treatment outcomes.

In our study, as delineated in Figure 2, we discerned a notable distinction in alpha diversity, while beta diversity remained relatively consistent. Alpha diversity primarily gauges the richness and evenness of species within a single sample. The marked difference suggests a variation in the number or distribution of microbial species within individual communities across different ethnic groups. On the other hand, beta diversity evaluates the dissimilarity between microbial communities from different samples. The lack of significant difference in beta diversity implies that while the individual communities might harbor varied species or their distributions, the overall microbial community structures across racial groups remain somewhat analogous.

This observation is of paramount importance. The pronounced difference in alpha diversity could be indicative of unique microbial species or strains that are predominant in one racial group but less prevalent or absent in others. Such microbial distinctions can potentially influence host metabolic activities, immune responses, and even drug metabolism, thereby impacting the efficacy and outcome of metastatic colorectal cancer treatments across different racial groups.

Our comprehensive investigation, driven by the objective of exploring racial variations in mCRC’s molecular and microbiological profiles, has unearthed pivotal insights. Notably, the intersection of microbiota and host genomics revealed the potential modulating effects of specific bacteria on gene expression. Of particular interest were the bacteria *Helicobacter* cinaedi and Sphingobium herbicidovorans and their correlated genes SELENBP1 and SNORA38, respectively. SELENBP1, or Selenium Binding Protein 1, has been increasingly recognized in oncology circles for its nuanced role in cancer biology (Pol et al., 2018). Several studies have postulated its role as a tumor suppressor. Reduced SELENBP1 expression has been linked to poor prognosis in several cancers, including lung and ovarian cancers (Huang et al., 2006; Wang et al., 2021). Its function is believed to be intricately linked with selenium; an essential trace element known to have anti-carcinogenic properties. SNORA38, on the other hand, is a part of the small nucleolar RNAs (snoRNAs) family, which primarily functions in the modification and processing of ribosomal RNA (rRNA) (Song et al., 2022). Emerging evidence suggests that dysregulation of snoRNAs can profoundly impact cellular homeostasis and potentially drive oncogenesis (Schulten et al., 2017). Particularly, SNORA38 has been identified as an oncogene in certain cancer types, playing a role in cellular proliferation and survival (Song et al., 2022). *Helicobacter* cinaedi, a bacterium traditionally associated with gastrointestinal infections, has recently been implicated in colorectal carcinogenesis (Liu et al., 2019). Its pro-inflammatory attributes potentially drive the inflammatory cascade, a recognized precursor to oncogenesis (Overacre-Delgoffe et al., 2021). Sphingobium herbicidovorans, though less studied, has its ties with xenobiotic degradation, which might have implications in carcinogen detoxification within the gut (Qiu et al., 2014). The final section of our study, focusing on the TCGA (COAD) data, reveals a remarkable pattern in the expression levels of SELEBP1 across different racial groups. This analysis underscores the nuanced interplay between genetics and race, particularly in the context of colorectal adenocarcinoma. The data unequivocally shows that SELEBP1 expression is lowest in White individuals and highest in Black or African American individuals, a finding that could have significant implications for personalized medicine and understanding racial disparities in cancer outcomes. However, it’s crucial to acknowledge the limitations of this study, primarily due to the disproportionate representation of racial groups in the sample. The notably lower number of Asian participants (N = 11) compared to Black or African American (N = 65) and White (N = 241) individuals may skew the interpretability and applicability of these findings to broader populations. This underrepresentation underscores a recurring challenge in genetic research: the need for more inclusive and diverse population samples to ensure that conclusions drawn are reflective of the global population.

In conclusion, our study underscores the intricate interplay between host genomics, actively transcribing tumor microbiota, and their collective role in mCRC pathogenesis. These findings can pave the way for a more personalized and racially tailored therapeutic approach, optimizing outcomes in the diverse mCRC patient population.

## Data Availability

The datasets presented in this study can be found in online repositories. The names of the repository/repositories and accession number(s) can be found in the article/Supplementary Material.

## Supplementary material

SUPPLEMENTARY FIGURE S1
**(A)** Heatmap illustrating differentially expressed genes unique to the White vs. Asian comparison. Further information on these genes is provided in Supplementary Table S3. **(B)** Heatmap depicting differentially expressed genes unique to the White vs. Black comparison. Detailed annotations are available in Supplementary Table S4. **(C)** Western blot analysis of SELENBP1 protein expression in colorectal cancer samples. Two distinct groups are represented: B (Black or African American) and W (White). The bands indicate the relative abundance of SELENBP1 protein in each group. Notably, the W group exhibits a significantly higher expression of SELENBP1 compared to the B group.
