# Supplementary figures and images for: Racial disparities in metastatic colorectal cancer outcomes revealed by tumor microbiome and transcriptome analysis with bevacizumab treatment
Source: Front Pharmacol. 2024 Jan 31;14:1320028. doi: 10.3389/fphar.2023.1320028 (PMC10864621; doi:10.3389/fphar.2023.1320028)

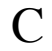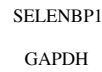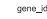

Supplement: Supplementary file 5 [file Image1.pdf]
